# Enhanced Catalysis of P-doped SnO_2_ for the V^2+^/V^3+^ Redox Reaction in Vanadium Redox Flow Battery

**DOI:** 10.3389/fchem.2021.688634

**Published:** 2021-06-24

**Authors:** Xiaojian Feng, Zixuan Zhang, Tongxue Zhang, Jing Xue, Chao Han, Lei Dai, Ling Wang, Zhangxing He

**Affiliations:** School of Chemical Engineering, North China University of Science and Technology, Tangshan, China

**Keywords:** vanadium redox flow battery, electrocatalyst, tin dioxide, P doping, energy storage

## Abstract

In this work, nanosized P-doped SnO_2_ (SnO_2_-P) was prepared by a sol–gel method as a catalyst for the V^3+^/V^2+^ redox reaction in vanadium redox flow battery. Compared with SnO_2_, the electrochemical performance of SnO_2_-P is significantly improved. This is because P doping provides more active sites and shows greatly improved electrical conductivity, thereby increasing the electron transfer rate. As a result, SnO_2_-P shows better catalytic performance than SnO_2_. The SnO_2_-P modified cell is designed, and it exhibits an increase of 47.2 mA h in discharge capacity and 8.7% in energy efficiency compared with the pristine cell at 150 mA cm^−2^. These increases indicate that the modified cell has a higher electrolyte utilization rate. This study shows that SnO_2_-P is a new and efficient catalyst for vanadium redox flow battery.

## Introduction

The excessive use of fossil fuels has resulted in an increasing depletion of energy resources and significant damage to the environment (Cheng et al., 2020; Huang et al., 2020; Liu et al., 2020; Li et al., 2021; Wang et al., 2021). Hence, the development of new energy sources for environmental protection is a critical issue (Wang T. et al., 2020; Chuanchang et al., 2020; Fang et al., 2020; He et al., 2020; Yang et al., 2020; Liu et al., 2021; Nie et al., 2021). These include green and clean energy sources, such as solar energy and tidal energy. Nevertheless, these energy sources have the disadvantage of poor stability and weak continuity (Wu et al., 2021; Zhang and Sun, 2021). Therefore, researchers must solve these problems by developing high-performance energy storage technology.

Skyllas-Kazacos et al. (1986) established the concept of vanadium redox flow battery (VRFB), which has advantages that include flexible design, environmental friendliness, and high reliability. VRFB currently represents a promising choice for energy storage challenges (Jelyani et al., 2016; Zhang et al., 2018; Jiang et al., 2021a; Ye et al., 2021). VO_2_^+^/VO^2+^ and V^3+^/V^2+^ solutions are designed as the positive and negative active species for VRFB, respectively (Jiang et al., 2021b; Lv et al., 2021). A H^+^ exchange membrane is placed in the middle to form a closed loop. The electrode is an important component of VRFB and is where the redox reaction takes place. Nowadays, carbon materials including graphite felt (GF), carbon cloth, and carbon felt are the main electrode materials for this technology. These materials have the advantages of large specific surface area, low cost, and good stability. However, their poor surface hydrophilicity and low electrochemical activity limit their application. Therefore, carbon-based materials need to be modified before usage.

The modification treatments are generally divided into two methods: direct activation and the introduction of catalysts. Direct activation, including acid and heat treatments, can improve the wettability of the material. The introduction of catalysts on the surface mainly includes metals, metal oxides, and carbon-based materials. The metals used include Ni, Pt (Tseng et al., 2013), and Ir (Wang and Wang, 2007). For example, González et al. (2011) studied the application of nano-Bi-modified GF for VRFB. The modified electrode showed excellent electrochemical performance and outstanding reversibility. This is due to the nano-Bi providing more active sites, which in turn promote the redox reaction. The typical metal oxides used include CeO_2_ (Zhou et al., 2014), Mn_3_O_4_ (Di Blasi et al., 2017), and TiO_2_ (Vázquez-Galván et al., 2019). For instance, Wu et al. (2014) prepared SbO_2_ modified GF by electrodeposition. Due to the excellent catalytic activity and stability of SbO_2_, the electrochemical performance of the cell was effectively improved. The carbon-based materials include carbon nanosheets (Wang J. et al., 2020), carbon nanotubes (Li et al., 2020), and carbon nanofibers (Wei et al., 2016). For example, Aziz et al. (2020) synthesized nitrogen-doped carbon nanorods (NCNR) *via* electrospinning. Compared with the original carbon felt, the carbon felt with NCNR had better rate capability. This was attributed to the significantly increased vanadium ion contact area and the accelerated electron transfer process.

SnO_2_ is a low-cost metal oxide with excellent electrochemical activity and is widely used in electrochemical fields (Liu et al., 2016; Ahmed et al., 2017; Chen et al., 2019; Zhang et al., 2020). Mehboob et al. (2018) reported that SnO_2_-deposited carbon felt had higher discharge capacity and cycle stability in VRFB. SnO_2_ was shown to have excellent electrocatalytic performance and was an efficient catalyst. However, the conductivity of SnO_2_ is poor, which limits its application. In order to solve this problem, P-doped SnO_2_ (SnO_2_-P) is prepared by a sol–gel method in this work. SnO_2_-P has better catalytic performance than SnO_2_. This is because SnO_2_-P combines the advantages of SnO_2_ and P. SnO_2_ mainly provides catalytic active sites, while the introduction of P improves the conductivity of the material. The electrochemical activity of the V^3+^/V^2+^ redox reaction in a VRFB is promoted. In summary, this work provides a potential method for improving the performance of VRFB using nanosized SnO_2_-P.

## Experimental

### Preparation of Materials

In this study, samples were prepared by a sol–gel method. SnCl_2_·2H_2_O (2.247 g) was weighed and dissolved into 20 ml of absolute ethanol. It was then also oscillated by ultrasound for 30 min and magnetically agitated at 65°C for 2 h in a water bath. When gradually forming the gel, 0.047 g of H_3_PO_4_ (85 wt.%) solution was added to SnO_2_ gel. The gel gradually turned pale yellow. The magnet was removed and sealed for aging for 24 h. The two samples were then dried at 80°C for 24 h in the vacuum drying oven and heated at 600°C for 2 h in a muffle furnace. The sample was named SnO_2_-P. Similarly, with SnCl_2_·2H_2_O particles as the tin source and phosphoric acid as the source of phosphorus doping, the solution was magnetically agitated to gradually make the gel and then to obtain SnO_2_.

### Characterization of Materials

To study the crystal phase of the samples, X-ray diffraction (XRD) was completed using a D/MAX2500PC instrument. Scanning electron microscopy (SEM, JSM-IT100) was used to analyze the morphology of the samples. The element composition and chemical state of the samples were studied by X-ray photoelectron spectroscopy (XPS) *via* a K-Alpha 1063 instrument (Thermo Fisher Scientific, United Kingdom).

### Electrochemical Measurements

The electrochemical measurements using an electrochemical workstation (CHI660E; Shanghai Chenghua Instruments) were performed by a system consisting of three electrodes. A glassy carbon electrode acted as the working electrode, a platinum electrode served as the counter electrode, and a saturated calomel electrode as the reference electrode. The 1 mg SnO_2_ sample was evenly mixed with 9 mg of acetylene black (AB), and the mixture was dispersed in N, N-dimethylformamide (DMF) under ultrasonic dispersion for 3 h. Finally, 20 μl dispersion completely fell on the electrode and it was then dried for 4 h at room temperature. The electrochemical measurements based on cyclic voltammetry (CV) and electrochemical impedance spectroscopy (EIS) were performed in a negative electrolyte of 1.6 M V^3+^ + 3.0 M H_2_SO_4_. The EIS test was performed in a constant potential mode. The frequency range was 10^−1^–10^6^ Hz.

### Charge–Discharge Tests

In a voltage window of 0.7–1.65 V, the charge–discharge performance of the cell was studied using a battery test device (CT 2001A; Wuhan Land, China). The 3 × 3 cm^2^ of GF, purchased from Jinglong Carbon Graphite Plant, was pretreated with ethanol for 30 min under the action of ultrasound. The ion exchange membrane (Nepem-1110) was pretreated by immersing in a 3.0 M H_2_SO_4_ solution over 24 h. This was used as the separation membrane for the positive and negative cells. SnO_2_-P (3 mg) was dispersed in 10 ml of DMF. The GF was then soaked in it for 3 h by ultrasonic dispersion to allow SnO_2_-P to be adsorbed on the GF. The modified GF was placed in the oven for drying. This process was used to prepare the modified GF, which was used as the negative electrode of the modified cell. For comparison, a pristine GF was used as the two electrodes of the pristine cell. To completely absorb the electrolyte, the pristine and modified GFs were soaked in the electrolyte of 0.8 M V^3+^ + 0.8 M VO^2+^ + 3.0 M H_2_SO_4_ for 12 h, respectively. The cell began a charge–discharge test three times to achieve an equilibrium of the positive and negative electrolytes at 10 mA cm^−2^.

## Results and Discussion

**FIGURE 1:**
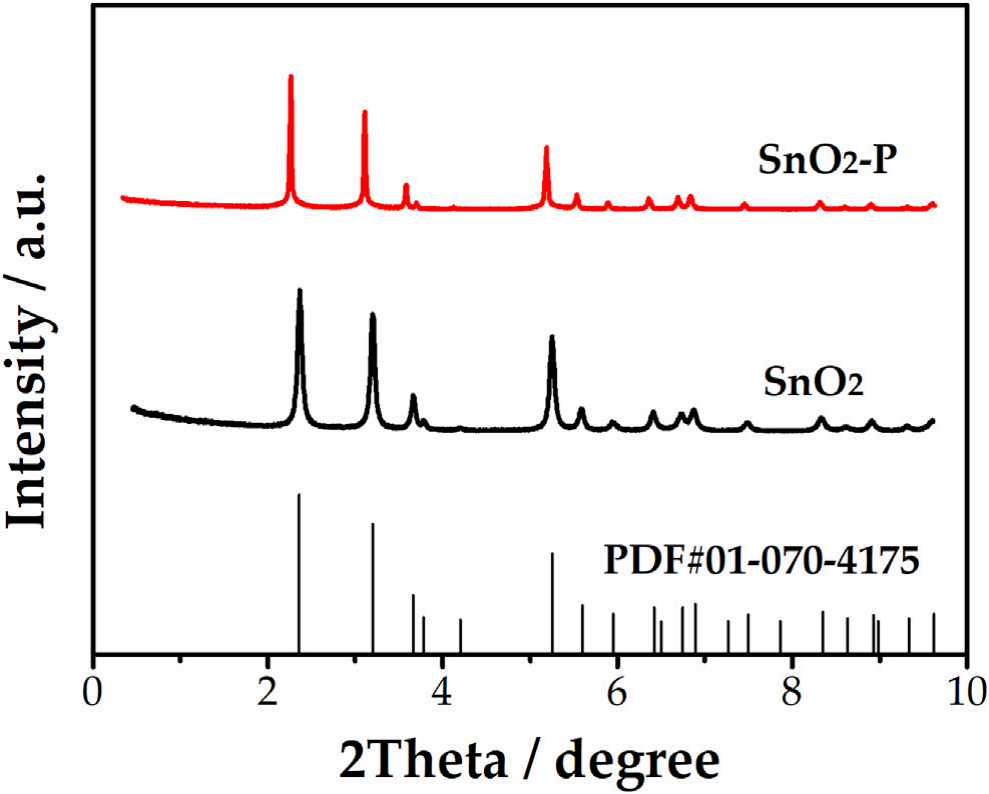
XRD patterns of SnO_2_ and SnO_2_-P.

Figure 1 shows the XRD patterns of SnO_2_ and SnO_2_-P. By comparing these XRD patterns, the qualitative identification of the phase composition and structure can be achieved. The characteristic peaks of SnO_2_ and SnO_2_-P are in the same place. The peak widths of the samples are close to each other. It can be seen that the observed peak corresponds to the standard value of SnO_2_ (JCPDC No. 01-070-4175). The structure of SnO_2_ corresponds to the tetragonal cassiterite type. There are no impurity characteristic peaks, indicating that no new phase is introduced by doping.

**FIGURE 2:**
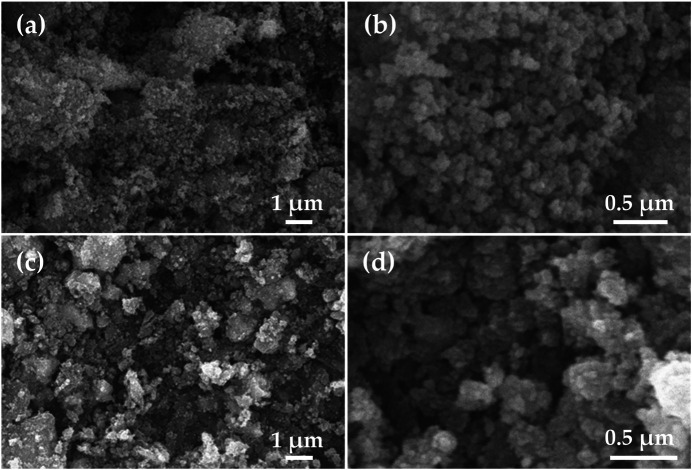
SEM images of SnO_2_ **(A**,**B)** and SnO_2_-P **(C**,**D)**.

Figure 2 shows the SEM characterization of SnO_2_ (Figures 2A,B) and SnO_2_-P (Figures 2C,D). The morphology of the SnO_2_ and SnO_2_-P nanoparticles was studied by SEM. The results show that the nanoparticles are uniformly dispersed without obvious agglomeration. There is no significant difference in the size of the nanoparticles after P doping, and the diameter of the two particles is between 200 and 300 nm. The aforementioned results show that P doping has no obvious effect on the morphology.

**FIGURE 3:**
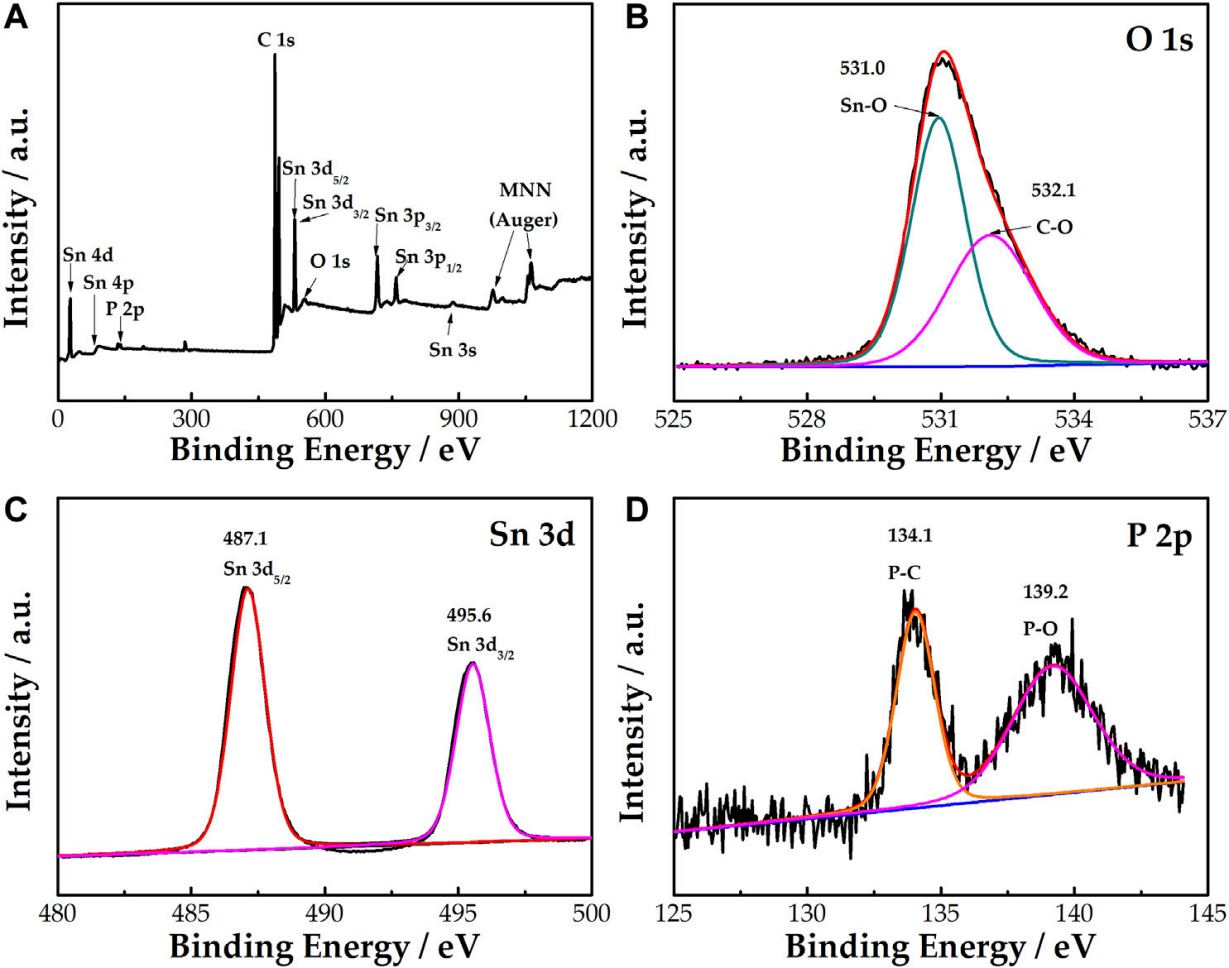
XPS survey spectrum of SnO_2_-P **(A)**. High-resolution XPS spectra of O 1s **(B)**, Sn 3d **(C)**, and P 2p **(D)** for SnO_2_-P.

Figure 3A shows that Sn, O, P, and C correspond to the binding energy table of elements. Combined with the previous XRD analysis, it can be confirmed that P has been doped into the SnO_2_ lattice. In Figure 3B, the O 1s peak can be convoluted into two peaks at 531.0 and 532.1 eV, representing the Sn-O and C-O bonds, respectively (Powell et al., 2018). Figure 3C indicates that there are two peaks at 495.6 and 487.1 eV corresponding to Sn 3d_3/2_ and Sn 3d_5/2_, respectively. Figure 3D indicates that there are two peaks at 134.1 and 139.2 eV, corresponding to the P 2p peak, thereby confirming the existence of P in the treated sample. The peaks of P at 134.1 and 139.2 eV represent the P-C and P-O bonds, respectively. The results show that Sn is only present in P^5+^, which replaces Sn ions to generate extra valence electrons, resulting in a decrease in resistivity.

**FIGURE 4:**
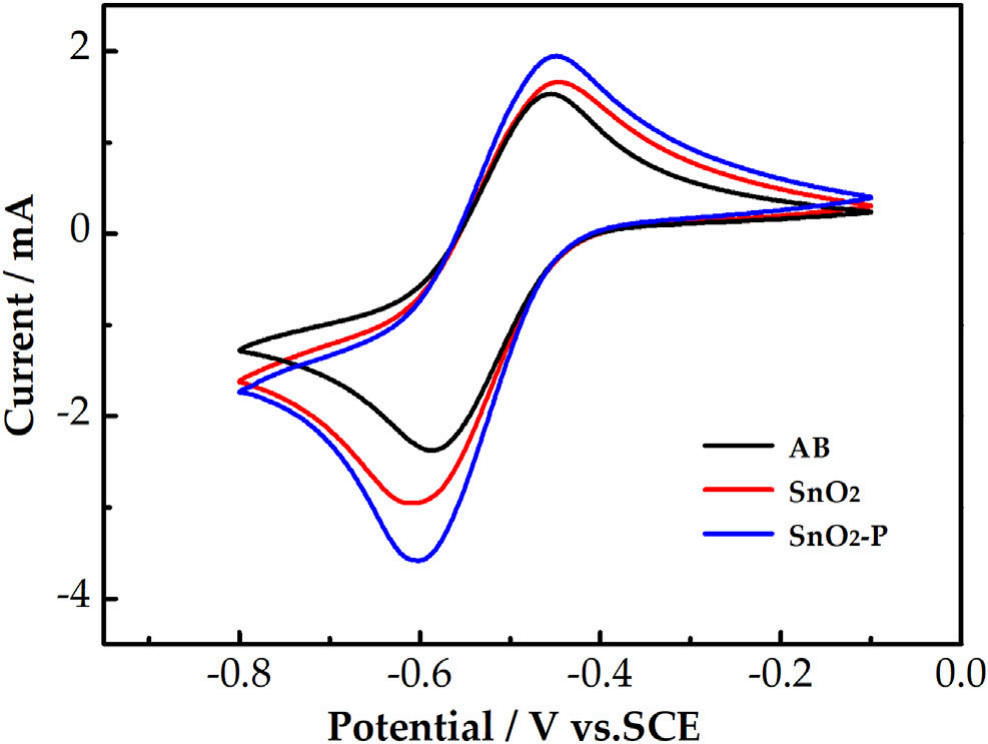
CV curves for AB, SnO_2_, and SnO_2_-P carried out in 1.6 M V^3+^ + 3.0 M H_2_SO_4_ electrolyte at a scan rate of 10 mV s^−1^.

The catalysis of AB, SnO_2_, and SnO_2_-P for the activity of the V^3+^/V^2+^ redox reaction was evaluated by CV measurements. The CV curves of the three samples are shown in Figure 4. All the electrodes have a higher reduction peak current than an oxidation peak current. This is due to the electrolyte containing more V^3+^ than V^2+^. The peak current of SnO_2_ (oxidation: 1.66 mA, reduction: 2.96 mA) is higher than that of AB (oxidation: 1.5 mA, reduction: 2.38 mA). This is due to SnO_2_ possessing good catalytic activity and providing active sites for the electrode reaction. Compared with that of SnO_2_, the peak current of SnO_2_-P is higher. This indicates that P doping improves the electrochemical properties of SnO_2_ for the redox reaction of the V^3+^/V^2+^ couple.

**FIGURE 5:**
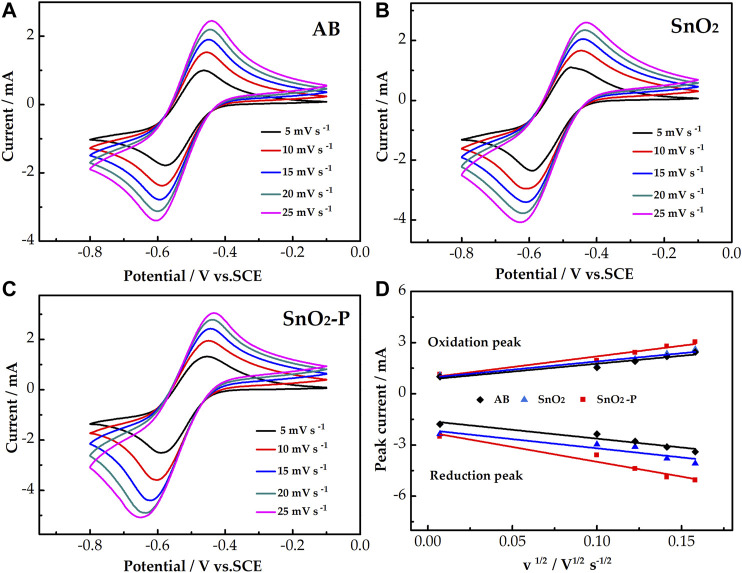
CV curves of AB **(A)**, SnO_2_ **(B)**, and SnO_2_-P **(C)** at scan rates from 5 to 25 mV s^−1^ carried out in 1.6 M V^3+^ + 3.0 M H_2_SO_4_ electrolyte. Plots of the redox peak current vs. the square root of the scan rate for the AB, SnO_2_, and SnO_2_-P electrodes **(D)**.

The CV test was carried out at different scan rates for further studying the influence of different electrodes on the mass transfer rate of the reactants. As presented in Figures 5A–C, for the three electrodes, the peak shape of all the CV curves remains symmetrical with an increasing scan rate. This proves that the electrochemical stability is good. In addition, the peak current increases as the scan rate increases. The oxidation and reduction peak potentials shift to the right and left, respectively. Figure 5D shows the relationship between the square root of the scan rate and the redox peak current. The peak current is obviously proportional to the square root of the scan rate for all samples. This proves that the redox reaction is under the control of the diffusion process. The mass transfer rate improves with an increase in the linear slope. The slope of SnO_2_ is larger than that of AB because SnO_2_ can provide more active sites. Simultaneously, the linear slope of SnO_2_-P is larger than that of SnO_2_. This indicates that P doping is beneficial to the migration of active substances.

**FIGURE 6:**
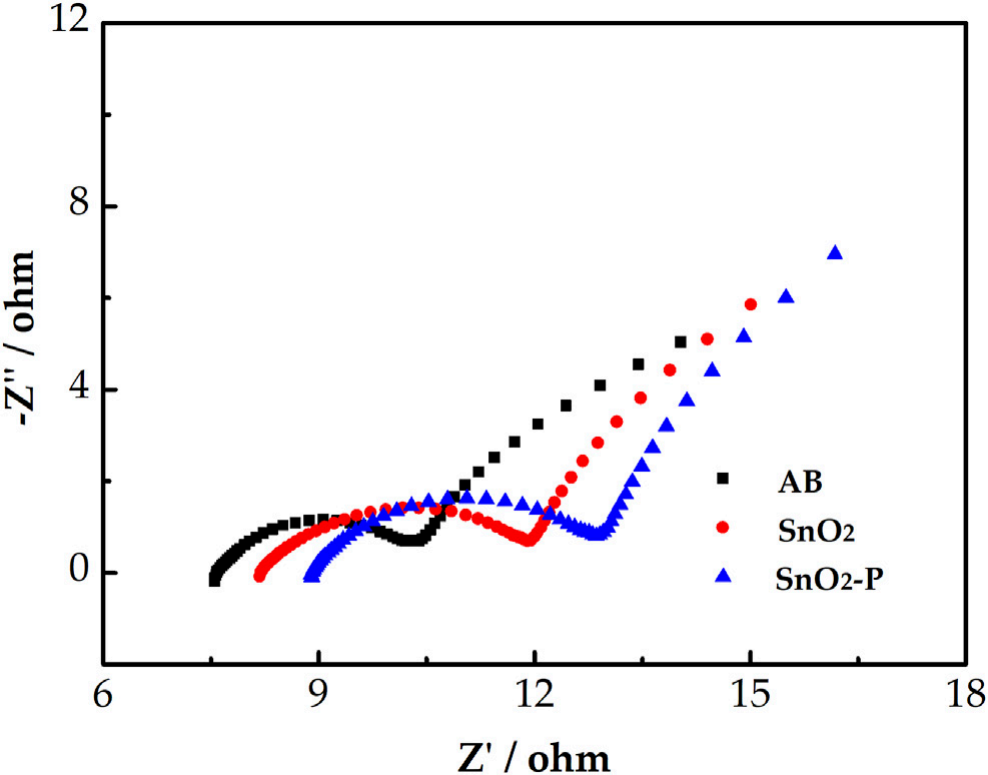
Negative Nyquist plots for AB, SnO_2_, and SnO_2_-P carried out in 1.6 M V^3+^ + 3.0 M H_2_SO_4_ electrolyte and the simplified electrical equivalent circuit fitting.

The EIS test was used to further investigate the electrocatalytic activity of the samples. Figure 6 shows the Nyquist diagram of the V^3+^/V^2+^ redox pair for the three electrodes. All Nyquist diagrams consist of a semicircle in the low frequency part and a slash in the high frequency part which are attributed to charge transfer and diffusion processes, respectively. An equivalent circuit was used to fit the Nyquist plots, where R_s_ is an ohmic resistance, including the solution, contact, and electrode resistance. R_ct_ represents the charge transfer resistance of the redox reaction. Q_m_ is a constant-phase element that reflects the double-layer capacitance in the electrode/electrolyte interface. The constant-phase element of Q_t_ corresponds to the ion diffusion capacitance.

Table 1 shows the corresponding fitting electrochemical parameters of the three samples. As shown, R_s_ follows the order of SnO_2_-P < SnO_2_ < AB, which indicates that SnO_2_-P exhibits the lowest ohmic resistance. In addition, R_ct_ displays the order of SnO_2_-P < SnO_2_ < AB. The smaller the R_ct_, the lower is the charge transfer resistance. The R_ct_ of SnO_2_ is smaller than that of AB. This is due to SnO_2_ having a certain catalytic activity and providing active sites. SnO_2_-P has the smallest R_ct_ because of the increase in electrode conductivity and charge transfer rate due to the P doping. The order of Q_t_ and Q_m_ is AB < SnO_2_ < SnO_2_-P. This is because P doping further increases the double-layer and diffusion capacitances, with the charge transfer and diffusion process promoted, respectively.

**TABLE 1:** Fitting of electrochemical parameters for AB, SnO_2_, and SnO_2_-P.

Sample	R_s_/Ω	Q_m_		R_ct_/Ω	Q_t_	
		Y_0_	n_0_		Y_1_	n_1_
AB	9.015	1.43 × 10^−2^	0.68	3.487	3.87 × 10^−5^	0.936
SnO_2_	8.317	1.837 × 10^−2^	0.65	3.234	5.84 × 10^−5^	0.891
SnO_2_-P	7.685	2.576 × 10^−2^	0.56	2.096	8.02 × 10^−5^	1.000

**FIGURE 7:**
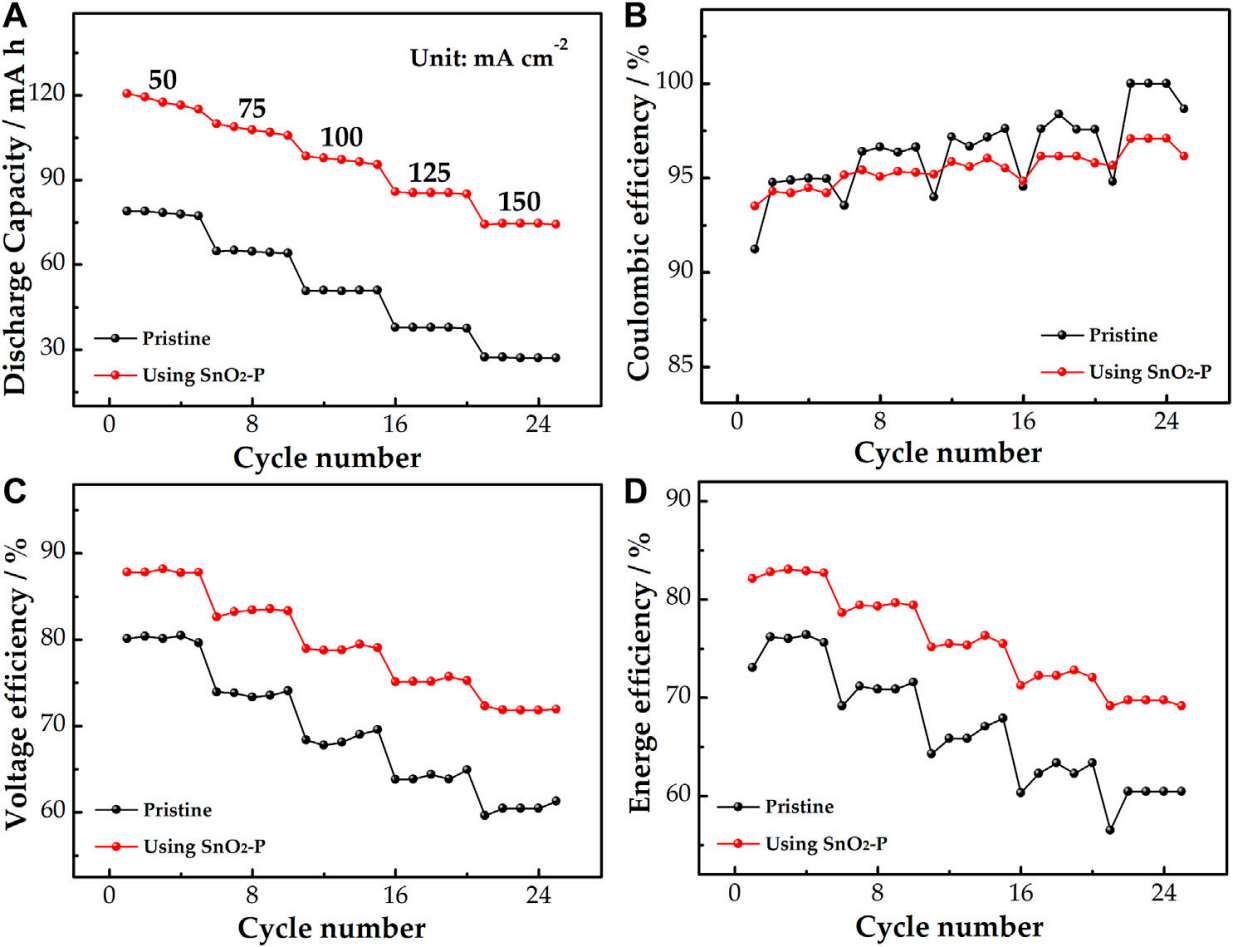
Discharge capacity **(A)**, CE **(B)**, VE **(C)**, and EE **(D)** of pristine and SnO_2_-P modified cells at a current density of 50–150 mA cm^−2^.

The rate performance of the pristine and SnO_2_-P modified cells was studied. As shown in Figure 7A, the discharge capacity of the cells reduces with increasing current density, which is because the high current density can produce electrochemical polarization. At 150 mA cm^−2^, the discharge capacity of the SnO_2_-P cell increases by 47.3 mA h from 27.1 mA h for the pristine cell. This shows that SnO_2_-P can improve the discharge capacity of the cell dramatically. Figure 7B presents the coulombic efficiency (CE), which is the ratio of discharge capacity to charge capacity. The CE can be used to reflect the degree of charge loss of cell. The CE using SnO_2_-P is marginally smaller than that of the pristine cell. It is due to a long charge–discharge time of the SnO_2_-P cell and serious infiltration of active substances.

As shown in Figure 7C, the voltage efficiency (VE) of the SnO_2_-P cell is increased in comparison with the pristine one. At 150 mA cm^−2^, the VE of the modified cell (71.9%) is increased by 10.6% compared to the pristine cell (61.3%), illustrating that SnO_2_-P effectively decreases the electrochemical polarization of the cell. As seen from Figure 7D, the energy efficiency (EE) is jointly determined by the VE and CE. The EE of the two cells decreases with increasing current density. During the whole charge–discharge process, the SnO_2_-P cell presents a higher EE than the pristine cell. The EE of the modified and pristine cells is 69.2 and 60.5% at 150 mA cm^−2^. This reveals that the SnO_2_-P cell has excellent energy storage capacity. The results show that the SnO_2_-P modified cell has good stability and high energy storage capacity, which can reduce the electrochemical polarization.

**FIGURE 8:**
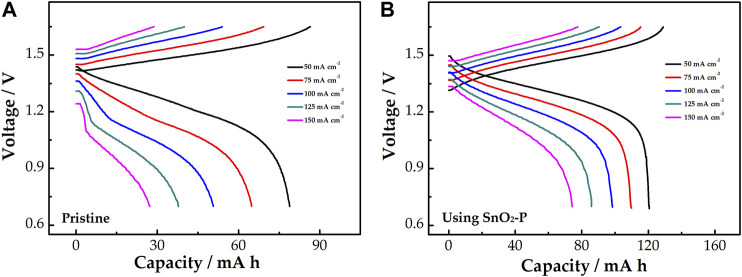
Charge–discharge curves of pristine **(A)** and SnO_2_-P modified **(B)** cells at a current density of 50–150 mA cm^−2^.

Figure 8 shows the relationship between the discharge capacity and voltage of the cells. The charge–discharge curves of pristine and modified cells under all current density are presented in Figures 8A,B, respectively. The discharge capacity gradually decreases with the current density increase under the same control voltage. Compared with the pristine cell, the SnO_2_-P cell has a lower charge voltage platform and a higher discharge voltage platform at the same current density, which means that it has a smaller charge–discharge voltage difference. This is because SnO_2_-P can decrease the electrochemical polarization of the cell. SnO_2_-P increases the mean discharge voltage of the cell, meaning that the energy density of the cell is also enhanced by SnO_2_-P.

## Conclusion

In this work, SnO_2_-P is a new catalyst for the V^3+^/V^2+^ redox pair in VRFB. SnO_2_-P has better electrocatalytic activity and kinetic reversibility than SnO_2_ and AB. This is because the P doping changes the original structure and thus has higher electrical conductivity, which increases the electron transfer rate of the vanadium redox reaction. The charge–discharge rate performance measurement at 50–150 mA cm^−2^ demonstrates that the cell using SnO_2_-P has a larger discharge capacity than the pristine cell, which indicates that the SnO_2_-P cell possesses a higher electrolyte utilization rate and better electrochemical stability. The VE and EE of the cell with SnO_2_-P are also greatly improved, indicating that SnO_2_-P can reduce the electrochemical polarization, and the energy density of the cell is improved. In conclusion, SnO_2_-P is a new type of VRFB catalyst with excellent potential.

## Data Availability

The original contributions presented in the study are included in the article/supplementary material; further inquiries can be directed to the corresponding authors.
